# Improved Pain and Function, Low Complication Rates, and 95% Patient Satisfaction at 2 Years After Medially Stabilized Total Knee Arthroplasty: A Prospective Multicenter Cohort Study

**DOI:** 10.1016/j.artd.2026.102051

**Published:** 2026-08-15

**Authors:** J. Baré, L. Bradley, R. Brighton, S. Talbot, D. Wood

**Affiliations:** a Melbourne Orthopaedic Group, Melbourne, Victoria, Australia; b Whangarei Hospital, Whangarei, New Zealand; c Westmead Private Hospital Sydney, Westmead, NSW, Australia; d Orthopaedic Department, Western Health, Melbourne, Victoria, Australia; e North Sydney Orthopedics and Sport Medicine Centre, Sydney, New South Wales, Australia

**Keywords:** Total knee arthroplasty, Medially stabilized, Multicenter, Prospective, Patient-reported outcomes

## Abstract

**Background:**

The medially stabilized (MS) knee concept has gained wider acceptance in recent years. However, to date, there is inconsistent evidence for its merits over traditional total knee arthroplasty (TKA) designs. The aim of this study was to assess the clinical performance of a second-generation medially stabilized knee obtainable by multiple, independent surgeons in a general TKA population.

**Methods:**

Fifteen surgeons (16 hospitals) participated in this prospective nonrandomized cohort study of 568 patients (608 knees). Demographics represented a standard primary TKA population. Data were collected for baseline characteristics and outcome measures at 6, 12, and 24 months postoperatively for range of motion, the Knee injury and Osteoarthritis Outcome Score, Oxford Knee Score, EuroQol 5-Dimension visual analog scale, University of California Los Angeles activity score, Forgotten Joint Score, patient satisfaction scores, and complications.

**Results:**

Overall, 548 TKAs were included in the final 2-year follow-up (90.8% follow-up). The revision rate at 2 years was 0.99% (6 knees revised). All measures reported significant improvement from preoperative to 24 months (*P* < .0001), with good range of motion (119°) and high mean scores, including Oxford Knee Score 42 and Forgotten Joint Score 70. At 2 years, the median satisfaction score was 9.3, 95.7% of patients reported that their problems were better and 94.5% of patients responding that the results of their operation were good, very good, or excellent.

**Conclusions:**

We found that use of a second-generation MS knee design by multiple surgeons can produce excellent outcomes, with low complication rates and higher levels of patient satisfaction, than those reported in the literature for traditional TKAs. This study represents the largest cohort, multisurgeon, prospective study reported in the literature for the introduction of a new generation MS TKA and supports its wider adoption.

## Introduction

The first medially stabilized (MS) total knee arthroplasty (TKA) was developed in the early 1990s to improve tibiofemoral stability [1]. Based on experience with preceding designs and observations of the native knee, the developers sought to achieve a stable articulation with a fully conforming medial ball-in-socket articulation and ligament balancing technique [1]. Axial rotation about the long-axis was permissible via a less-constrained lateral condyle [1]. After its introduction, magnetic resonance imaging studies confirmed the observed medial stability in the native knee with axial tibial rotation exhibited by translation on the lateral side [[2], [3], [4]]. Subsequently the MS concept was shown to reproduce a similar kinematic profile to the native knee [5] and promising patient outcomes were reported [1,[6], [7], [8]].

Despite its theoretical potential, wider acceptance of the MS knee concept has been limited. Inconsistent outcomes for devices with the MS concept [[9], [10], [11], [12]] and the success of traditional designs are likely to have contributed to this. Nevertheless in 2022, the annual report of the Australian Joint Replacement Registry reported use of the concept had grown in the recent decade to 10% of all primary TKAs in Australia [13]. With growing interest in the concept and with the introduction of new MS knee implants, there is a need to assess the evidence for its reliability and any vulnerabilities regarding patient selection or its wider adoption.

Using complications data and patient-reported outcome measures (PROMs), the aim of this study was to assess the clinical performance of a second-generation MS knee design when introduced to general use by multiple surgeons who had not been involved in developing the device and did not previously use a MS knee. To the authors’ knowledge this is the only large cohort, prospective, multicenter outcomes study reported in the literature evaluating the introduction of a second-generation MS knee design to treat a normal demographic of TKA patients, independent of designer surgeons and surgeons with prior experience of using the concept. It was hypothesized that high levels of function and patient satisfaction would be observed when assessing the 2-year outcomes of this second-generation MS design.

## Materials and methods

This prospective large cohort, nonrandomized, multicenter study was approved by the Human Research Ethics Committees at the St Vincent’s Hospital, Melbourne (HREC-D 151/15), The Avenue Hospital, Melbourne (HREC 194) and the Whangarei Hospital, Whangarei (Northland District Health Board 2015-29), with site approvals obtained from the participating hospitals. Between January 2016 and May 2020, 608 TKAs (568 patients) were performed by 15 surgeons in 16 sites using the SAIPH Knee (MatOrtho Ltd., Leatherhead, UK). The design of the prosthesis is characterized by asymmetry between compartments, with the medial side of the prosthesis a single radius, spherical component congruent with the medial tibial polyethylene component through a full range of motion (ROM). The lateral side is not congruent allowing anteroposterior translation in addition to rotation. Changes in the second generation of the prosthesis were confined to the nonarticular portion of the prosthesis to improve anatomical replication and bone preservation. Adult individuals requiring a primary knee arthroplasty were invited to participate in the study. Indications for the use of the prosthesis were determined by the treating surgeon and aligned to the manufacturer’s instructions for use. The exclusion criteria included pregnancy or planning on becoming pregnant during the follow-up period for women; individuals with local or systemic infection; severe neurological, vascular or muscular deficiencies; individuals with severe bone loss or collateral ligament instability. Invited patients were provided with the patient information sheet and consent form at the surgical booking consultation and provided an opportunity to ask questions of the surgeon. Patients returned a signed consent form at the time of booking or later by mail or at a subsequent consultation prior to surgery. Surgeons were selected on the basis that they were all established knee arthroplasty surgeons but had only recently started using this prosthesis. No surgeon was involved in the design or had any commercial interest in the prosthesis. All surgeons received a full introduction to the concept, the device and fundamental surgical principals prior to use. No additional training specific to the use of the prosthesis was required. Each surgeon had performed at least five cases with the prosthesis prior to patient recruitment. All devices were cemented, the posterior cruciate ligament (PCL) was fully excised in all cases and coronal balance in flexion and extension was achieved either through soft tissue release or bony alignment strategies depending upon the individual surgeon’s preference. Indeed, common variations in surgeons’ established practice, such as the threshold for patella resurfacing, were deemed to constitute an acceptable representation of general use.

**Figure 1:**
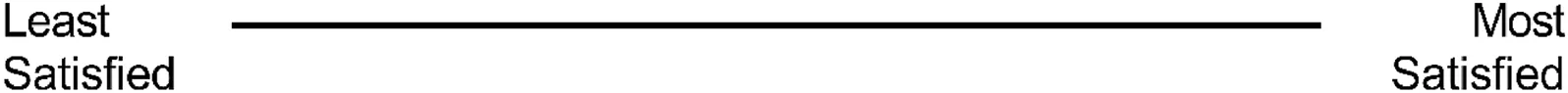
Analog VAS satisfaction scale: patients were asked to mark the line at the position that best represents their level of satisfaction with their TKA.

Demographic data included age, gender, side, date of operation, body mass index, indication for surgery, and follow-up length. The cohort demographics, indications for surgery, application of alignment strategy, and patellar resurfacing were indicative of standard TKA practice in the participating hospitals (Table 1). PROMs included the Knee injury and Osteoarthritis Outcome Score (KOOS), the Oxford Knee Score (OKS), the EuroQol 5-Dimension visual analog scale (VAS), the University of California Los Angeles activity score, the Forgotten Joint Score (FJS), and patient satisfaction scores. Patients were asked “Overall, how are your problems now, compared to before your operation?” with a 5-point Likert response. Patients were also asked “How would you describe the results of your operation?” on a 5-point Likert response as previously described [14], as well as a 100-mm VAS (with “least satisfied” at the left end and “most satisfied” at the right end of the line) that corresponded with how satisfied they were with their knee arthroplasty overall (Fig. 1). Patients were clinically evaluated preoperatively and at 6 weeks, 6, 12, and 24 months postoperatively, with any complications and all-cause revisions recorded at the time of reporting. The ROM and PROMs were collected preoperatively and at 12 and 24 months postoperatively.

**Table 1:** Summary of patient demographics, indication for surgery, and intersurgeon variability.

Parameter	Value
Mean age ± SD (range)	68 ± 8 (38-92)
Gender, male:female % (no.)	49.8:50.2 (303:305)
Surgical side, left:right % (no.)	43.8:56.3 (266:342)
Mean BMI ± SD, kg/m^2^ (range)	31.4 ± 6.5 (15.4-78.5)
Mean follow-up ± SD, months (range)	25.4 ± 2.2 (20.1-41.7)
Indication for surgery, % (no.)	
Osteoarthritis	95.6 (581)
Inflammatory arthritis	2.0 (12)
Osteonecrosis	0.8 (5)
Trauma	0.5 (3)
Other	0.8 (5)
Not specified	0.3 (2)
Alignment, % (no.)	
Mechanical alignment	91.4 (556)
Modified kinematic alignment	8.6 (52)
Instrumentation (alignment referencing), % (no.)	
Intramedullary femur, extramedullary tibia	42.7 (260)
All intramedullary	29.8 (181)
Patient-specific (image derived instrumentation)	21.4 (130)
Navigation	4.9 (30)
Other	0.2 (1)
Not specified	1.0 (6)
Patella resurfacing, % (no.)	
Resurfaced	65.6 (399)
Not resurfaced	34.0 (207)
Not specified	0.4 (2)

BMI, body mass index; SD, standard deviation.

Participating surgeons were required to perform uniform face-to-face consultations for patients preoperatively and at 6 weeks and 12 and 24 months postoperatively, in alignment with the study protocol. At each of these reviews, the surgeons collected PROMs and clinical data on a standardized form. All collected study information was then forwarded to a centralized data collection. All data were recorded onto the Socrates Orthopaedic Outcomes Software (Ortholink, PTY Ltd., Sydney, Australia).

### Data analyses

Statistical analysis was performed using the GraphPad Prism software (version 9.3.1, December 2021). Datasets were assessed for normality using the Shapiro-Wilk test. Nonparametric Mann-Whitney or Kruskal-Wallis tests with post hoc Dunn’s multiple comparisons were used to compare ROM and PROMs data at each time point. Data are reported as mean ± standard deviation with 95% confidence interval (CI) or min-max range. The Kaplan-Meier method was used to evaluate implant survivorship. The threshold for significance for all statistical analyses was *P* < .05.

## Results

Outcome scores were available at the 2-year time point for 548 TKAs (516 patients, 90.8%). Five patients (5 TKAs, 0.8%) had died from unrelated causes, 9 (9 TKAs, 1.5%) had withdrawn from the study, 36 (40 TKAs, 6.6%) had been lost to follow-up, and 6 (6 TKAs, 0.99%) had been revised.

**Figure 2:**
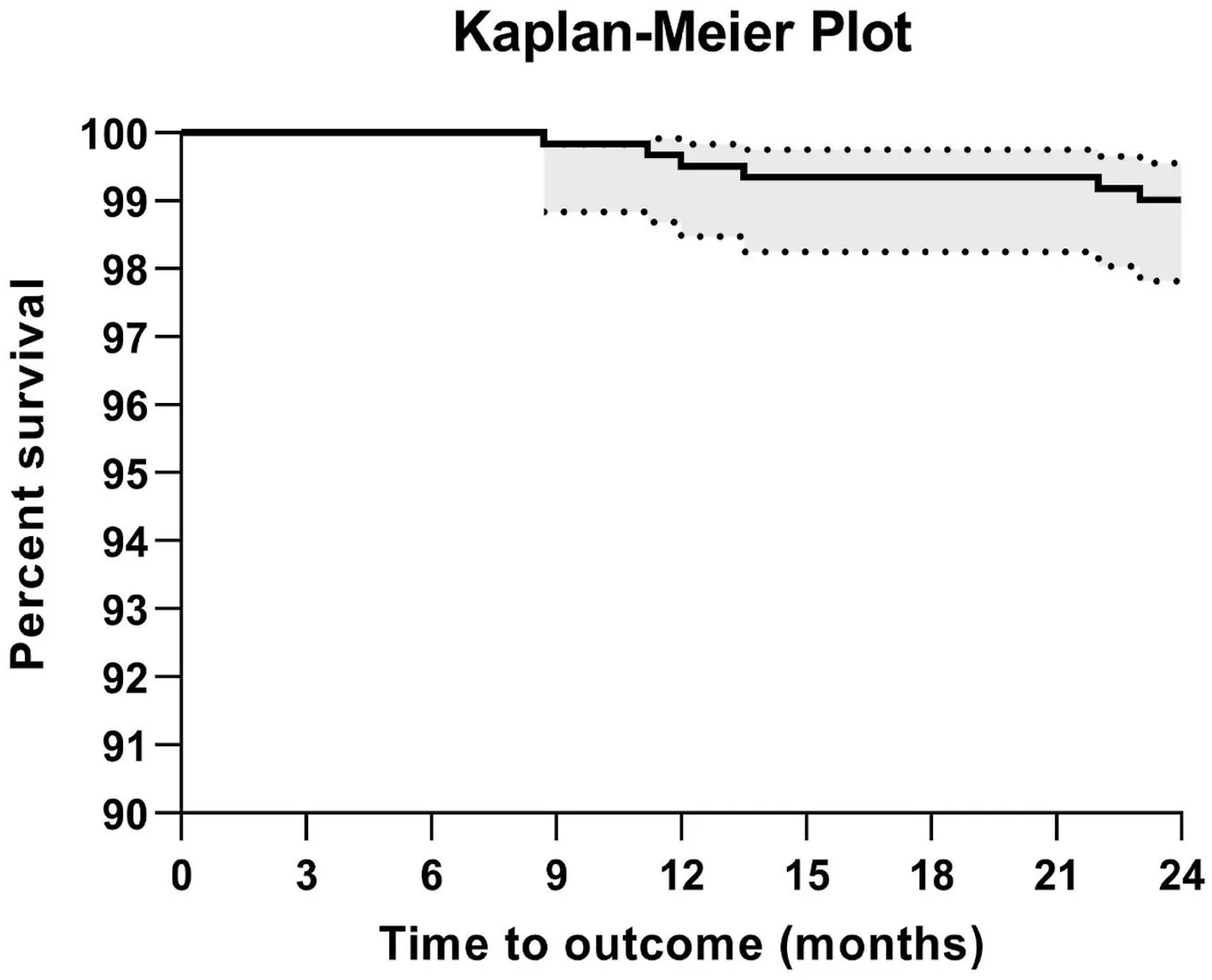
Kaplan-Meier survival curve for all-cause revision within cohort across 2-year follow-up.

**Figure 3:**
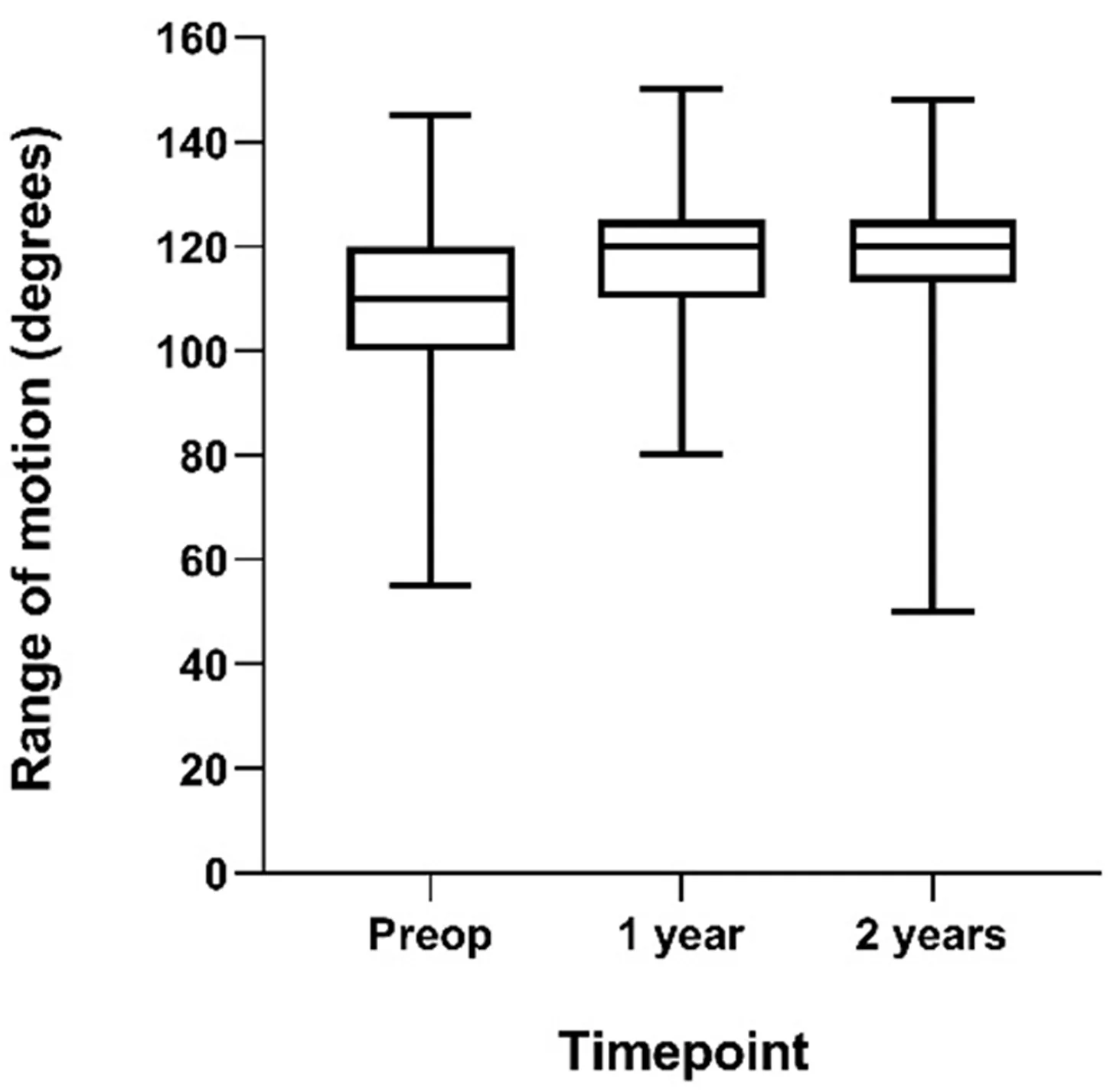
Boxplot (mean, interquartile range, range) of maximum ROM assessed preoperatively and at 1 and 2 years postoperatively.

The overall revision rate was 0.49% (95% CI 0.16-1.52) at 1 year and 0.99% (95% CI 0.44-2.18) at 2 years (6 of 608 TKAs revised) (Fig. 2). Reasons for revision included general instability (N = 1), stiffness (N = 1), patella resurfacing (N = 1), valgus malalignment (N = 1), and infection (N = 2). Stiffness requiring manipulation under anesthetic was reported in 14 cases (2.3%). At the 2-year follow-up time point, 17 cases (2.8%) exhibited a flexion range less than 100°, of which 3 cases (0.5%) were still under clinical review. The mean (range) ROM for the cohort improved from 110° (55-145) preoperatively to 118° (80-150) at 1 year (*P* < .001) and 119° (50-148) at 2 years (*P* < .0001) (Fig. 3). There was no significant difference in ROM at 1 and 2 years postsurgery (*P* = .095). Significant improvements were seen from presurgery to 2 years for all scores, and between 1 and 2 years for the OKS, KOOS symptoms, KOOS quality of life, and the FJS (Table 2).

**Table 2:** Summary of patient-reported outcomes—values reported as mean ± SD (range), *number of knees*.

Score	Preoperative	1-y postoperative	2-y postoperative	*P* value^a^	
				Preoperative – 2 y	1 y-2 y
KOOS symptoms	45 ± 19 (0-93) *608*	81 ± 16 (14-100) *549*	84 ± 14 (14-100) *544*	<.0001	0.041
KOOS pain	45 ± 17 (0-100) *608*	86 ± 16 (17-100) 549	89 ± 14 (22-100) *546*	<.0001	0.122
KOOS ADL	50 ± 19 (0-99) *607*	88 ± 14 (24-100) *545*	90 ± 14 (18-100) *545*	<.0001	0.063
KOOS QoL	25 ± 18 (0-88) *608*	73 ± 21 (0-100) *549*	77 ± 21 (0-100) *543*	<.0001	0.033
KOOS sports	18 ± 21 (0-100) *598*	59 ± 30 (0-100) *494*	62 ± 30 (0-100) *483*	<.0001	0.577
FJS	-	64 ± 29 (0-100) *522*	70 ± 28 (0-100) *542*	-	<0.0001^b^
OKS	21 ± 8 (2-45) *608*	40 ± 7 (9-48) *548*	42 ± 7 (8-48) *544*	<.0001	0.03101
UCLA	4 ± 2 (1-10) *605*	6 ± 2 (1-10) *549*	6 ± 2 (1-10) *543*	<.0001	>0.999
EQ-5D VAS	69 ± 20 (10-100) *605*	82 ± 16 (22-100) *550*	81 ± 17 (6-100) *548*	<.0001	>0.999

ADL, KOOS Activities of Daily Living subscale; EQ-5D, EuroQol 5-dimension; QoL, KOOS Quality of Life subscale; UCLA, University of California Los Angeles activity score; SD, standard deviation.

Italic values indicate number of knees.

a Kruskal-Wallis with post hoc Dunn test (specified if different).

b Mann-Whitney test.

**Figure 4:**
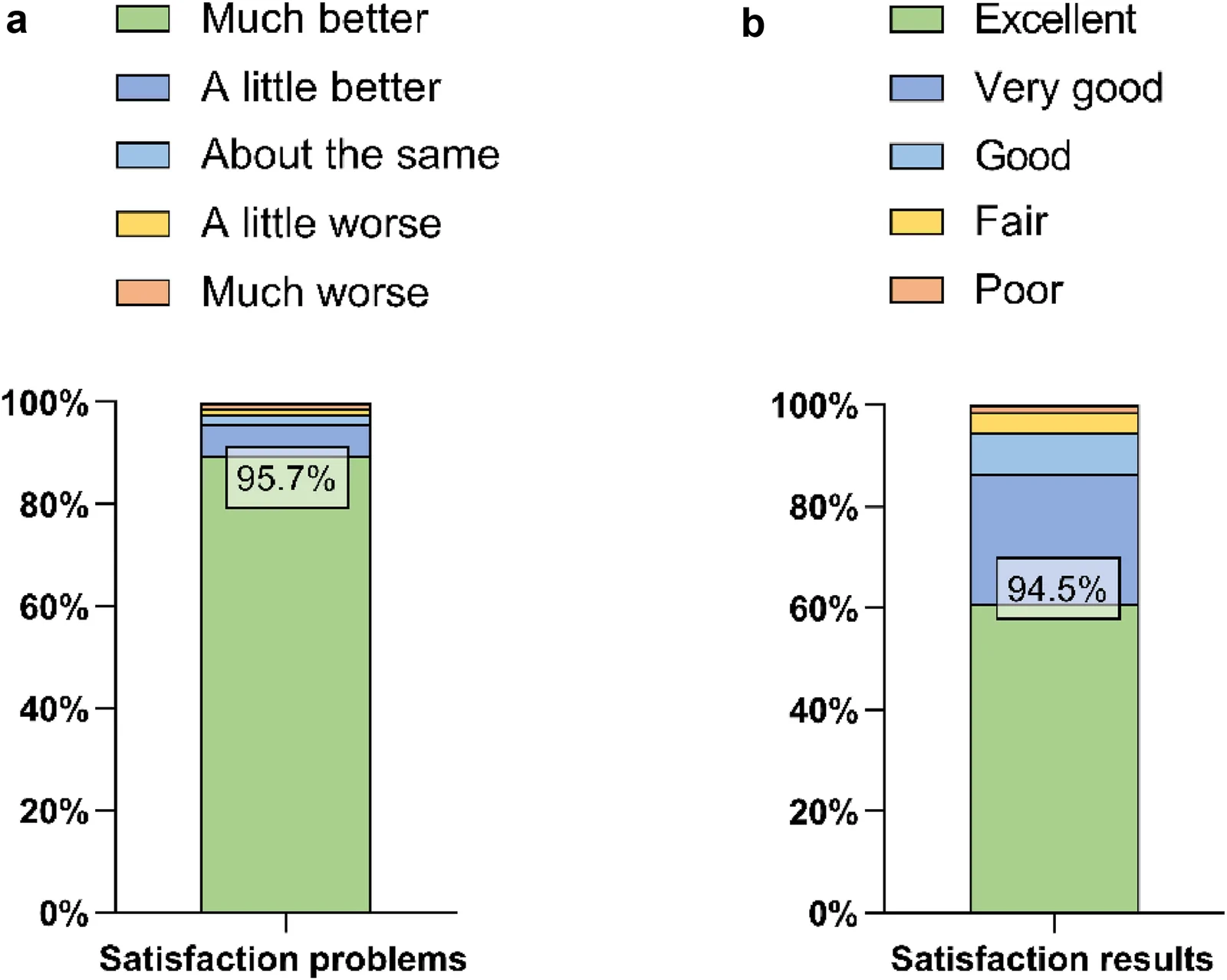
Satisfaction outcomes at 2 years postsurgery answering the questions (a) “Overall, how are your *problems* now, compared to before your operation?” and (b) “How would you describe the *results* of your operation?”.

**Figure 5:**
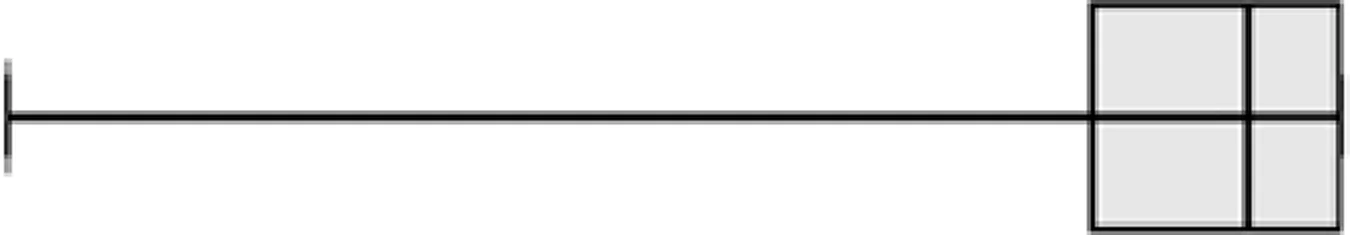
Illustrative boxplot (mean, interquartile range, range) produced using patient responses on the VAS satisfaction scale.

When asked “Overall, how are your problems now, compared to before your operation?” 95.7% of patients answered “much better” or “a little better” at 2 years postsurgery. When asked “How would you describe the results of your operation?” 94.5% of patients answered “good,” “very good,” or “excellent” at 2 years (Fig. 4). The median satisfaction VAS was 9.2 at 2 years (IQR 8.2-10). Figure 5 illustrates patients’ responses at the 2-year time point.

The potential learning curve was assessed using the OKS, ROM, satisfaction score, and revisions for the first 10 and following 40 procedures for all surgeons who performed at least 50 operations (8 surgeons; 80 cases in the “first 10” group and 320 cases in the “last 40” group). No statistically significant differences were seen between these groups for the OKS, ROM, or satisfaction scores at either 1- or 2-year follow-up (Table 3).

**Table 3:** Summary of values of the outcome measures considered for the evaluation of surgeon learning curve—values reported as mean ± SD (range).

Parameter	First 10 cases		Last 40 cases		*P* value^a^ 1 y	*P* value^a^ 2 y
	1 y	2 y	1 y	2 y		
OKS	41 ± 7 (10–48)	41 ± 9 (10–48)^a^	41 ± 6 (16–48)	42 ± 6 (16–48)	0.946	0.874
ROM, *degrees*	117 ± 11 (80–150)	119 ± 14 (50–145)^a^	118 ± 11 (87–145)	120 ± 11 (85–148)	0.301	0.712
Satisfaction score	8.9 ± 1.5 (4.0–10.0)	8.7 ± 2.0 (2.0–10.0)^a^	8.9 ± 1.5 (2.0–10.0)	8.9 ± 1.7 (0.0–10.0)	0.845	0.133
Revisions, *n*	1 patella resurfacing (12 mo)	1 infection (22 mo)	1 stiffness (11 mo)	None	-	-

a Mann-Whitney test.

## Discussion

MS bearings in TKA have received increasing attention as an evolutionary modification to fixed bearings to better replicate native tibiofemoral kinematics. While the concept has undergone a storied history in its implementation through various products introduced to market from the mid-1990s [12], contemporary systematic reviews have identified comparable midterm performance to standard fixed bearing designs in prosthesis survival and patient-reported outcomes [15]. The evaluation of this second-generation MS device (SAIPH Knee) builds on its first-generation predecessor, the medial rotation knee introduced in 1994, which demonstrates relatively low revision rates at 10-year follow-up (2.56%) [16]. The staged introduction of the present device has verified the medial pivot kinematics of the implant [17] and returned encouraging clinical outcomes in a designer-surgeon series [18]. Subsequent investigations by nondesigner surgeons reported improved clinical knee stability (N = 60) [19] and superior clinical results (N = 46) [20] over contemporary fixed-bearing devices.

All TKA designs have sought to provide stability, longevity, and normal kinematics and the MS knee concept has achieved these aims [13,17,19]. Measures of success are now more aligned to the patient’s perception with goals to reduce the proportion of patients who are unsatisfied with their TKA. By comparing PROMs and satisfaction among patients with MS and non-MS knee designs, Jones et al. [19] concluded knee stability had the greatest influence on patient outcome measures. Pritchett reported that patients can notice the difference and cite stability as a reason for preferring a MS knee over posterior stabilized and cruciate retaining (CR) knees [7]. However, wider literature [[9], [10], [11], [12],^15^] has shown that inclusion of a medial ball-and-socket articulation in a TKA design does not guarantee improved outcomes. In our study, we evaluated whether the introduction of a second-generation MS knee design to wider use by multiple surgeons could produce consistently high outcomes as reported by the patients. While our study did not assess differences between different MS knee designs and constructs, or the techniques employed to implant them, all surgeons used the same device that had been developed based on experience with the first-generation design [17], with good education prior to use, complete excision of the PCL, and availability only of cemented components. The present analysis represents a definitive multicenter evaluation of this prosthesis in a nondesigner series and the largest cohort study on this category of bearing, comparable to all pooled studies to-date (N = 630) [21]. In line with the hypothesis, the results demonstrated high levels of postoperative function and patient satisfaction, as well as reasonably low joint pain and awareness at 2-year follow-up.

Complications recorded in our study were comparable to literature that attributes most complications to patient factors and pre- and postoperative care [22]. In our study, special attention was given to complications citing stiffness, given the additional constraint in the implant design and surgeons’ limited experience with the concept. In the present series, one case was revised for stiffness, and 2.3% of cases were treated for stiffness by manipulation under anesthetic. Overall, the median ROM at 2 years (119°) was comparable to the average of previous medial-pivot (PCL-sacrificing) designs (117°) [21] and within the range for a broad collection of implant designs at final follow-up [9], as well as the upper boundary of a reference trajectory after TKA [23]. Nevertheless, a proportion of patients (2.3%) reported <100° of maximum flexion at the 2-year follow-up, which is a lower incidence than reported in a localized registry at up to 6 months of follow-up (18.5%) [22]. The MS design is intended to be implanted with more ligament laxity in flexion than contemporary CR designs, without loss of stability [20], and may be less forgiving of a tight flexion gap. Attempts to retain the PCL have been also shown to result in poorer outcomes [19]. Thus, complete and thorough resection of the entire PCL and more physiological ligament laxity, particularly on the lateral side in flexion, are prerequisites for optimal balancing of this TKA, which may translate to a learning curve for surgeons practiced in CR knee designs. We found that using the study device and with appropriate training, increased rates of stiffness were avoided. The relatively few occurrences of stiffness recorded in this study did not represent a measurable learning curve.

Indeed, there was no detectable learning curve within any measure in this study. Although the literature tends to focus on operative time as a convenient indicator of surgeon efficiency during uptake of a new device or technique modification [24], it does not represent the important indicators of patient outcome. There is a risk of increased rate of revision in cases performed as part of the learning curve [25], as well as higher complication rates [26] following the introduction of a new device; however, this was not observed in the present series. The lack of discernible differences in subsamples representing different parts of the surgeon learning curve align with other larger studies examining the learning curve after the introduction of a new implant into practice [27]. In their large cohort study (N = 2000), Whittaker et al. [27] detected clinically insignificant differences for PROMs, including the KOOS Activities of Daily Living subscore and no differences for complications. Given these findings, surgeons taking up the present implant of interest may expect an increase in operative time initially, but a general lack of learning curve with respect to patient-centered outcomes. However, larger samples to adequately power learning curve analyses for complications and revisions are required.

The outcomes in this patient cohort are within the reported population variability for pain, function, joint awareness, as well as revision endpoints. It should be noted that the Australian Orthopaedic Association National Joint Replacement Registry population for the device in question overlaps with the present cohort, as well as representing broader usage beyond the staged introduction. The upper confidence limit (95%) of the revision rate at 2-year follow-up in the present series (2.18%) is higher than the limit at 3 years for the same device (2.0%) in Australia [13] and its predecessor design at 3 years in the UK population (1.27%) [16], but this may be at least partly attributable to the differences in sample sizes. Further, the upper limit in the present series is below the upper confidence limit reported for other medial pivot devices (2.5-3.9%) [13]. Initial concerns that the asymmetric constraint in MS TKA might lead to an increase in tibial loosening were alleviated in midterm radiological and outcomes evaluation of the first-generation implant [1]. Previous reviews of revision and failure modes in medial pivot designs in multiple registries worldwide [10] have also identified implant loosening as a key reason for revision; this is in contrast to the lack of revisions for loosening (femur or tibia) in our series.

Patient-reported knee-localized pain and function, as derived from the OKS and KOOS, were comparable to results for broader TKA populations in national registry reports from New Zealand, Australia, Sweden, the United Kingdom, and the United States. The mean OKS score at 1 year in the present study (40) is in line with the 6-month means reported by the New Zealand Joint Registry (37.8) [28], Australian Joint Replacement Registry (37.6) [29], and 1-year results from the Dutch Arthroplasty Register (39.1) [30]. Similarly, the mean KOOS-Pain subscale (86) at 1 year was comparable to the average for 65-74-year-olds (84.8) observed in a primary knee arthroplasty multicenter registry in the United States (N = 3539) [31] and the Swedish Arthroplasty Register at 1-year (78) [32]. Further, the change in physical activity rating (University of California Los Angeles activity score) of 2 points is in line (1.9 points) with reports for patients >55 years [33] after primary TKA.

Behrend et al. [34] introduced the FJS as a measure of patients’ ability to forget their joint in everyday life, proposing it to be the ultimate goal to ensure maximum patients’ satisfaction. Their TKA patients reported a FJS of 50, whereas healthy control subjects of the same age reported a FJS for the knee of 71.7. With a FJS of 70 at 2 years after surgery, this large multicenter cohort achieved a mean FJS representative of the healthy control subjects reported by Behrend et al. Such scores after TKA have been reported elsewhere [35,36], but it is notable that when compared to their standard practice CR knees, three of the surgeon study participants reported a higher FJS for this MS knee at the 1-year time point [19,20]. This is consistent with findings of a recent meta-analysis reporting a significantly greater FJS for MS knees than non-MS knees [11]. Tso et al. [11] also found the FJS to represent the greatest difference among PROMs scores between knee types. Regarding our observed improvement from 1 to 2 years, Carlson et al. reported similar improvements over the same period but found that the FJS later declined (76.4-64.4) from 2 to 5 years postoperation [36]. With the designer series for this device reporting a high FJS of 75.3 at 5 years [18], follow-up of this cohort to 5 years would be of interest.

While the FJS reflects functional outcome, patient satisfaction after TKA is multifactorial and is influenced by expectations and factors beyond function [37]. Contemporary literature suggests an average dissatisfaction rate after primary TKA of 10%, which remains a high proportion of patients, but may vary in relation to the incidence of known risk factors within a given population [37], such as obesity [38]. The average body mass index observed in this study places the cohort in the obese (class I) category. Nevertheless, good-excellent satisfaction rates at 2-year follow-up (94.5%) compared favorably to a localized Australian registry (N = 2226) at 6-month follow-up (89%) [39], which also identified a potential relationship between osteoarthritis severity and likelihood of dissatisfaction after primary TKA. The present results also compare favorably to multiple studies of satisfaction after TKA (89-96.2%) [14] with these rates expected to be stable over medium to long-term follow-up. The high level of patient satisfaction in our cohort is consistent with the designer series for this MS knee [18].

The findings should be interpreted within the scope and limitations of the study. The findings demonstrate positive outcomes out to 2-year follow-up, and these results are expected to be stable into medium term follow-up [14]. However, further monitoring is necessary to identify late-term issues. The present results should also be viewed within the context of biases that are prevalent in observational studies of this kind. The influence of selection bias on the part of surgeons (patient selection) and the patients themselves (accepting the study and implant) cannot be ruled out entirely; however, the direction and magnitude of its effect on the results cannot be determined with the information available. Surgeon bias was somewhat mitigated by some surgeons using pseudorandomization techniques to select patients for inclusion in the study [20], the consecutive manner of recruitment overall, and the multicenter study design. Some of the analyses (eg, for learning curve) may have been constrained by a lack of power and should be interpreted with some caution. Meaningful comparisons to benchmarks for complications and revision incidence require larger samples to test hypotheses with available methods. Nevertheless, the results demonstrate that this MS knee design can produce consistently good clinical results when used by multiple surgeons in their general practice. The strengths of this study were the large cohort of patients evaluated, and the number of surgeons and hospitals involved. Despite intrinsic differences in prior experience and standard practice among participating surgeons, clinical outcomes for the SAIPH Knee were consistent and comparable with previously published literature for the same device including those for the designer surgeon series [18], supporting its wider adoption.

## Conclusions

In this large, multicenter, multisurgeon study using a second-generation MS knee design, irrespective of surgeons’ prior experience of the concept or preferred planning and alignment strategies, patients consistently reported excellent PROMs outcomes and higher levels of satisfaction than is reported in the literature. We conclude that sound application of the MS knee concept represents a meaningful contribution to the advancement of state-of-the-art TKA and a step forward in our endeavors to meet contemporary patient expectations.

## CRediT authorship contribution statement

**J. Baré:** Writing – review & editing, Writing – original draft, Visualization, Supervision, Resources, Project administration, Methodology, Investigation, Data curation, Conceptualization. **L. Bradley:** Writing – review & editing, Writing – original draft, Methodology, Investigation, Data curation. **R. Brighton:** Writing – review & editing, Writing – original draft, Methodology, Investigation, Data curation. **S. Talbot:** Writing – review & editing, Writing – original draft, Methodology, Investigation, Data curation. **D. Wood:** Writing – review & editing, Methodology, Investigation, Data curation.

## Conflict of interest

J. Baré receives funding from MatOrtho Ltd for support for the present manuscript including study conduct and payment of article processing charges, payment of speaker fees for presentations at company educational events, support for attending conferences and meetings, and declares a leadership role as president of the Arthroplasty Society of Australia; L. Bradley is a Mathys medical advisor and is a member of the New Zealand Orthopaedic Association Education Committee; S. Talbot receives payment of speaker fees from Medacta and MatOrtho Ltd and is a paid consultant for MatOrtho Ltd.; D. Wood receives payment of speaker fees from MatOrtho SAIPH Knee, is a MatOrtho paid consultant, holds shares in Allegra Orthopaedics Limited, is a *American Journal of Sports Medicine* reviewer, and is a past president and secretary of the Australian Knee Society; all other authors declare no potential conflicts of interest.

For full disclosure statements refer to https://doi.org/10.1016/j.artd.2026.102051.

## Appendix A Supplementary data

Conflict of Interest Statement for Baré

Conflict of Interest Statement for Bradley

Conflict of Interest Statement for Brighton

Conflict of Interest Statement for Talbot

Conflict of Interest Statement for Wood
