# Supplementary material for: Improved Pain and Function, Low Complication Rates, and 95% Patient Satisfaction at 2 Years After Medially Stabilized Total Knee Arthroplasty: A Prospective Multicenter Cohort Study
Source: Arthroplast Today. 2026 Aug 15;40:102051. doi: 10.1016/j.artd.2026.102051 (PMC13499489; doi:10.1016/j.artd.2026.102051)
Supplement: Supplementary file 1 [file mmc1.docx]

# INDIVIDUAL CONFLICT OF INTEREST STATEMENT

***American Association of Hip and Knee Surgeons***

(Adopted from the American Academy of Orthopaedic Surgeons disclosure statement)

The following form **must be filled out completely and submitted by each author (example, 6 authors, 6 forms).**

**All items require a response. If there is no relevant disclosure for a given item, enter "*None*.”**

**Manuscript Title**

1.	Royalties from a company or supplier (The following conflicts were disclosed): Corin

2.	Speakers bureau/paid presentations for a company or supplier (The following conflicts were disclosed): Corin, MatOrtho, Medacta

3A.	Paid employee for a company or supplier (The following conflicts were disclosed): Nil

3B.	Paid consultant for a company or supplier (The following conflicts were disclosed): Corin, MatOrtho, Medacta

3C.	Unpaid consultants for a company or supplier (The following conflicts were disclosed): Nil

4.	Stock or stock options in a company or supplier (The following conflicts were disclosed): Nil

5.	Research support from a company or supplier as a Principal Investigator (The following conflicts were disclosed): MatOrtho

6.	Other financial or material support from a company or supplier (The following conflicts were disclosed): Nil

7.	Royalties, financial or material support from publishers (The following conflicts were disclosed): Nil

8.	Medical/Orthopaedic publications editorial/governing board (The following conflicts were disclosed): Nil

9.	Board member/committee appointments for a society (The following conflicts were disclosed): Nil

**Each author must sign AND print or type his/her name, date and submit a separate form**

In addition, one BLINDED Conflict of Interest form (no author names used) should be submitted per manuscript with all author disclosures.

Jonathan Baré	22 July 2026

Author Name (Print or Type)	Author Signature	Date
