# Supplementary material for: Improved Pain and Function, Low Complication Rates, and 95% Patient Satisfaction at 2 Years After Medially Stabilized Total Knee Arthroplasty: A Prospective Multicenter Cohort Study
Source: Arthroplast Today. 2026 Aug 15;40:102051. doi: 10.1016/j.artd.2026.102051 (PMC13499489; doi:10.1016/j.artd.2026.102051)
Supplement: Supplementary file 3 [file mmc3.docx]

# INDIVIDUAL CONFLICT OF INTEREST STATEMENT

***American Association of Hip and Knee Surgeons***

(Adopted from the American Academy of Orthopaedic Surgeons disclosure statement)

The following form **must be filled out completely and submitted by each author (example, 6 authors, 6 forms).**

**All items require a response. If there is no relevant disclosure for a given item, enter "*None*.”**

**Manuscript Title:** Improved Pain and Function, as well as Low Complications Incidence and 95% Patient Satisfaction with a Medially Stabilised Total Knee Arthroplasty Observed in a Prospective, Multicentre Cohort Study with 2-year Follow-up

1.	Royalties from a company or supplier (The following conflicts were disclosed)

NONE

2.	Speakers bureau/paid presentations for a company or supplier (The following conflicts were disclosed)

NONE

3A.	Paid employee for a company or supplier (The following conflicts were disclosed)

NONE

3B.	Paid consultant for a company or supplier (The following conflicts were disclosed)

NONE

3C.	Unpaid consultants for a company or supplier (The following conflicts were disclosed)

NONE

4.	Stock or stock options in a company or supplier (The following conflicts were disclosed)

NONE

5.	Research support from a company or supplier as a Principal Investigator (The following conflicts were disclosed)

NONE

6.	Other financial or material support from a company or supplier (The following conflicts were disclosed)

NONE

7.	Royalties, financial or material support from publishers (The following conflicts were disclosed)

NONE

8.	Medical/Orthopaedic publications editorial/governing board (The following conflicts were disclosed)

NONE

9.	Board member/committee appointments for a society (The following conflicts were disclosed)

NONE

**Each author must sign AND print or type his/her name, date and submit a separate form**

In addition, one BLINDED Conflict of Interest form (no author names used) should be submitted per manuscript with all author disclosures.

R Brighton	**Roger Brighton	19/06/24**

Author Name (Print or Type)	Author Signature	Date
