# Supplementary material for: Improved Pain and Function, Low Complication Rates, and 95% Patient Satisfaction at 2 Years After Medially Stabilized Total Knee Arthroplasty: A Prospective Multicenter Cohort Study
Source: Arthroplast Today. 2026 Aug 15;40:102051. doi: 10.1016/j.artd.2026.102051 (PMC13499489; doi:10.1016/j.artd.2026.102051)
Supplement: Supplementary file 5 [file mmc5.docx]

**INDIVIDUAL CONFLICT OF INTEREST STATEMENT**

***American Association of Hip and Knee Surgeons***

(Adopted from the American Academy of Orthopaedic Surgeons disclosure statement)

## The following form must be filled out completely and submitted by each author (example, 6 authors, 6 forms). All items require a response. If there is no relevant disclosure for a given item, enter *"None."*

**Manuscript Title:** Improved Pain and Function, as well as Low Complications Incidence and 95% Patient Satisfaction with a Medially Stabilised Total Knee Arthroplasty Observed in a Prospective, Multicentre Cohort Study with 2-year Follow-up

1.	Royalties from a company or supplier (The following conflicts were disclosed)

2.

Speakers bureau/paid presentati ns for a?omwany or supplier (The following conflicts were disclosed)

None

# f

Presentations for MatOrtho Saiph Knee

3A.

Paid employee for a company or supplier (The following conflicts were disclosed)

None

3B.	Paid consultant for a company or supplier (The following conflicts were disclosed)

MatOrtho

3C.	Unpaid consultants for a company or supplier (The following conflicts were disclosed)

None

4.

Stock or stock options in a company or supplier (The following conflicts were disclosed)

Shares in Allegra Orthopaedics Limited

5. Research support from a company or supplier as a Principal Investigator (The following conflicts were disclosed)

None

6. Other financial or material support from a company or supplier (The following conflicts were disclosed)

None

7. Royalties, financial or material support from publishers (The following conflicts were disclosed)

None

8. Medical/Orthopaedic publications editorial/governing board (The following conflicts were disclosed)

Reviewer for American Journal of Sports Medicine

9. Board member/committee appointments for a society (The following conflicts were disclosed)

Past President and Secretary of Australian Knee Society

Date

## Each author must sign AND print or type his/her name, date and submit a separate form

In addition, one BLINDED Conflict of Interest form (no author names used) should be submitted per manuscript with all
